# Optimizing 2D gas chromatography mass spectrometry for robust tissue, serum and urine metabolite profiling

**DOI:** 10.1016/j.talanta.2017.01.003

**Published:** 2017-04-01

**Authors:** Zhanru Yu, Honglei Huang, Alexander Reim, Philip D. Charles, Alan Northage, Dianne Jackson, Ian Parry, Benedikt M. Kessler

**Affiliations:** a Target Discovery Institute, Nuffield Department of Medicine, University of Oxford, Roosevelt Drive, Oxford OX3 7FZ, UK; b Shimadzu UK Limited, Mill Court, Featherstone Road Wolverton, Mill South, Milton Keynes MK12 5RD, UK

**Keywords:** Two dimensional gas chromatography, Mass spectrometry, Electron impact spectrum, Metabolomics, Bio fluid, Tissue cell extract

## Abstract

Two-dimensional gas chromatography mass spectrometry (GCxGC-MS) is utilized to an increasing extent in biomedical metabolomics. Here, we established and adapted metabolite extraction and derivatization protocols for cell/tissue biopsy, serum and urine samples according to their individual properties. GCxGC-MS analysis revealed detection of ~600 molecular features from which 165 were characterized representing different classes such as amino acids, fatty acids, lipids, carbohydrates, nucleotides and small polar components of glycolysis and the Krebs cycle using electron impact (EI) spectrum matching and validation using external standard compounds. Advantages of two-dimensional gas chromatography based resolution were demonstrated by optimizing gradient length and separation through modulation between the first and second column, leading to a marked increase in metabolite identification due to improved separation as exemplified for lactate versus pyruvate, talopyranose versus methyl palmitate and inosine versus docosahexaenoic acid. Our results demonstrate that GCxGC-MS represents a robust metabolomics platform for discovery and targeted studies that can be used with samples derived from the clinic.

## Highlights

• GCxGC-MS detected ~600 features;165 represented metabolites of different classes.
• Optimizing gradient length and separation through modulation improved metabolite ID.
• improved separation of lactate/pyruvate, talopyranose/palmitate and inosine/docosahexaenoate.

## Graphical abstract

**Figure:**
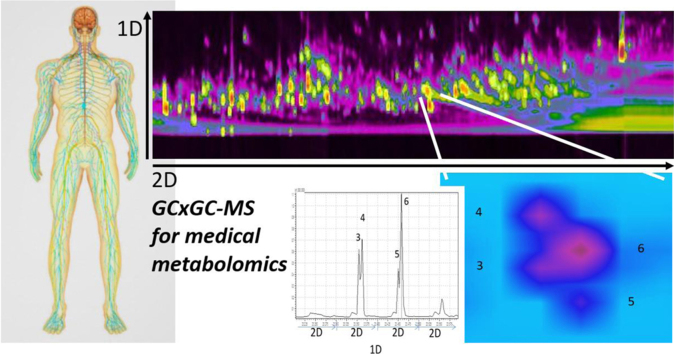

## 1 Introduction

Profiling metabolic processes in cellular physiology has been the driving force for analytical method development since decades. In recent years, metabolic reprogramming of cells has gained considerable attention in the context of cancer, but also immune cell biology [1], [2]. One of the hallmarks of metabolic changes upon an exogenous stimulus triggering activation and proliferation has been characterized as the Warburg Effect, describing a switch to glycolysis rather than oxidative phosphorylation driven cellular energy production [3]. This metabolic principle has been observed in highly proliferative cancer cells, but also a number of other cell types that respond to external stimuli via induction of proliferation. This phenomenon revived interest in metabolic processes, in particular underlying clinical pathology, and has accelerated advances in comprehensive analytical detection of metabolites [4]. The most frequently used techniques to measure metabolites are nuclear magnetic resonance spectroscopy (NMR) and liquid (LC) or gas chromatography (GC) coupled to mass spectrometry (MS) [5], [6], [7]. Metabolites have polar or nonpolar and organic as well as inorganic properties. These various traits make their separation very complex as there is no single analysis platform that can separate and detect all of these molecules at once. GC-MS has been particularly useful for the analysis of low-polarity volatile metabolites of fats and esters, but also high-polarity molecules such as amino- and organic acids that need to be converted into volatile derivatives [8], [9], [10], [11]. Limitations in resolving power of GC-MS have been improved by the development of two-dimensional workflows (GCxGC) [12] combined with high-speed scan single quadrupole (qMS) [13], [14], [15], [16], [17] and high-resolution mass spectrometry (GCxGC-TOF MS) [18], [19], [20], [21], [22]. In a GCxGC configuration, two columns with different properties (apolar vs polar) are connected through a modulator, allowing further separation of compounds that co-elute from the first column, thereby giving rise to enhanced resolution and peak capacity. Technically, this has been achieved by either closed cycle refrigerated loop modulation [23] or flow based modulation [24], [25]. Challenges in the analysis of complex GCxGC-MS data has so far precluded the use of this approach for the analysis of complex biological samples, but more recent developments in GCxGC-MS workflows and software tools have now made this approach more feasible for metabolomics experiments including biomarker discovery [19], [22], [26], [27]. In this study, we describe a robust GCxGC-qMS platform applied to the profiling of a panel of metabolites covering different chemical classes present in clinically relevant samples such as mammalian cells, tissue, serum and urine.

## 2 Experimental

### 2.1 Chemicals and metabolic standards

Chloroform and *tert*-butyl methyl ether were purchased from Fisher Scientific. Methanol and methoxyamine hydrochloride were from Sigma-Aldrich. N-methyl-N-trimethylsilyltrifluoroacetamide (MSTFA) with 1% chlorotrimethylsilane (TMCS) was purchased from Thermo Scientific. Metabolic standards used in this study were purchased from Sigma Aldrich with the exception of the following ones: cholesterol and pyridine (Alfa Aesar), creatinine (Acros Organics), myristic acid-14,14,14-d_3_ used as an internal standard (Cambridge isotope Laboratories) and *N*-oleoyl glycine (Cayman Chemical).

### 2.2 Tissue, serum/plasma, urine and cell lines used in this study

Rat kidney tissue material was kindly provided by Dr. Zee Akhtar [28] under the animal/ethic license PPL 30/2750. Aliquots of serum/plasma and urine derived from pigs were kindly provided by Professor. Benthe Jesperson (University of Ahus, Denmark) approved by the Danish National Animal Ethics Committee (no. 2008-561-1584.) U2OS and T24 cells were cultured in DMEM medium supplemented with 10% FCS and 1% penicillin/streptomycin at 37 °C.

### 2.3 Experimental methods

#### 2.3.1 Metabolite extraction from cell lines, tissue, serum and urine samples

Extraction of metabolites was carried out at room temperature if unspecified. For fluid samples, 200 µl of serum, plasma or urine were mixed with 200 µl of methanol and 5 µl myristic acid-14,14,14-d_3_ (1 mg/ml). The samples were vortexed for 5 min after adding 1 ml of *tert*-butyl methyl ether (MTBE). The suspensions were shaking for 5 min and centrifuged for 20 min at 13,000*g* at 4 °C. The organic phase (MTBE) was transferred to a glass vial and dried in a Speed Vac. Subsequently, 800 µl methanol was added into the aqueous remains, the suspension was vortexed for 5 min and centrifuged for 20 min at 13,000*g* at 4 °C. The supernatant (aqueous) was collected and added into the glass vial containing the organic phase to dry under vacuum. The dried samples were kept at −80 °C until use.

5 mg Tissue or 5×10^6^ cells were used for the extraction of metabolites. Tissue material or harvested cells were washed twice with ice cold PBS, resuspended in ice cold methanol and H_2_O (1:1 v/v) (400 µl) and 5 µl myristic acid-14,14,14-d_3_ (1mg/ml), and crude extracts were transferred into a bead beater tube containing washed glass beads (same volume as cell pellet/tissue piece). Samples were subsequently homogenized in a bead beater (Precellys 24, Bertin Technologies) for four cycles (6500 Hz, 45 s), followed by the addition of 1 ml of tert-butyl ether (MTBE) to extract metabolites. After vortexing for 5 min and centrifugation for 20 min at 13,000*g* and 4 °C, the organic phase was transferred to a glass vial and dried by Speed Vac centrifugation. To the remaining aqueous phase, 800μl of methanol was added, samples homogenized for one cycle (6500 Hz, 45 s), kept at −80 °C for one hour and centrifuged for 20 min at 13,000*g* and 4 °C. 1 ml of aqueous phase was added to the glass bead vial containing the organic phase and the samples dried in vacuo (Speed Vac Centrifugation).

#### 2.3.2 Chemical derivatization

Chemical derivatization was performed essentially as described [29]. In brief, samples were resuspended in a solution of 20 μg/µl methoxyamine hydrochloride in pyridine (50μl/sample) and shaken (1200 rpm) for 90 min at 30 °C. 70 µl N-Methyl-N-trimethylsilyltrifluoroacetamide (MSTFA) with 1% chlorotrimethylsilane (TMCS) and 30 µl pyridine were added to the samples, followed by incubation for one hour at 60 °C at a shaking speed of 1200 rpm. The samples were cooled down to temperature ambient and injected directly for GC-MS analysis.

#### 2.3.3 GCxGC-MS analysis

The samples were immediately analyzed using a GCxGC-MS system comprising of a gas chromatograph coupled to a quadrupole mass spectrometer (Shimadzu GCMS QP2010 Ultra) and a Shimadzu AOC-20i/s auto sampler as described [17]. The first dimension separation was carried out on a SHM5MS capillary column (30 m×0.25 mm i.d.×0.25 µm film thickness, Shimadzu) while the second dimension separation was on a BPX-50 capillary column (5 m×0.15 mm i.d.×0.15 µm film thickness, SGE). Helium gas was used as a carrier gas at a 73 psi constant inlet head pressure. The modulation period was set as 6 s. The samples were injected at 280 °C in different split ratios (between 1:1 to 1:200). The oven temperature was programed from 60 °C to 320 °C at 10 °C/min unless stated otherwise and held at 320 °C for 8 min. The interface temperature to the mass spectrometer was set at 330 °C and ion source was heated at 230 °C. The MS was operated at scan speeds between 5000 and 20,000 amu covering a range of m/z 45–600. Electron Ionization spectra were recorded at 70 eV.

#### 2.3.4 Data processing and analysis

Raw GCxGC MS data were processed using GCMSsolution software (v2.72/4.20 Shimadzu), and Chromsquare software (v2.1.6, Shimadzu) and GC Image (v2.3) in combination with the NIST 11/s, OA_TMS, FA_ME and YUTDI in-house libraries were used for data analysis. The annotation of metabolites was carried out by comparing them to external standards (IM spectra and retention times adjusted to the internal standard myristic acid-14,14,14-d_3_) and by spectrum matching based searches with the above databases for those metabolites without external standards. The similarity score threshold was set to 80 (out of 100), and the confidence of identification further validated by manual inspection of matches between experimentally observed and reference EI spectra. In case those detected peaks (blobs) were assigned to more than one metabolite (all scores above 80), only the highest score assignment was reported. For peak picking and peak quantitation using the GCMS Solution software (v4.2), we used the following parameters: i) for 1D-GC-qMS data: Slope: 100/min, width: 2 s, min area 20,000, drift 0/min and T. DBL: 1000 min without any smoothing methods used; ii) for 2D-qMS data: Width: 0.2 s, min area 20,000, drift 0/min and T. DBL: 1000 min without any smoothing methods used. For the samples using different injection ratios, we adjusted the slope/min parameter as follows: injection ratio (slope/min) 0.5/200 (7200), 1/200 (22,000), 1/100 (22,000), 1/40 (22,000), 1/20 (22,000), 1/10 (68,000), 1/5 (230,000), 1/1 (440,000) (Table S1). Limit of detection (L.O.D) values were calculated based on the following equation: L.O.D. (L_D_)=3.3xσ/S, where σ is the standard deviation observed for the analyte at a quantifiable concentration and S is the slope of the calibration curve [30], [31].

## 3 Results and discussion

### 3.1 GCxGC-qMS covers a wide range of clinical metabolites

**Fig. 1:**
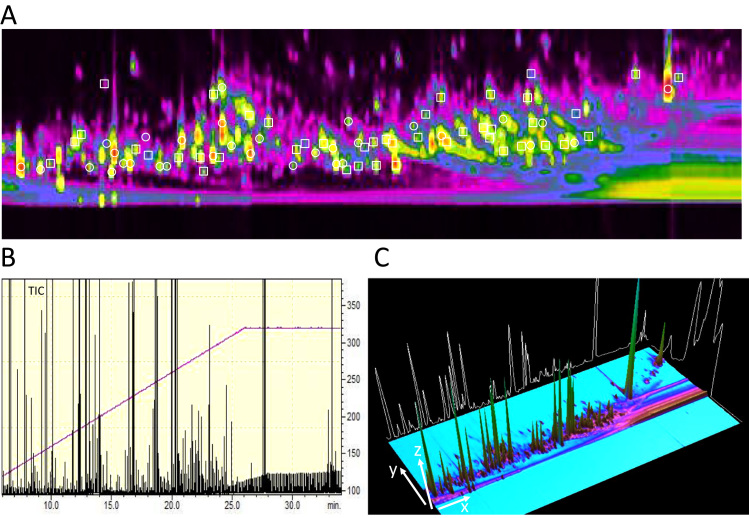
***Comprehensive GCxGC-MS profiling of cancer cell extracts.*** (**A**) 2D-GC map showing separation and detection of 614 molecular features in cell extracts, from which metabolites were identified either by database matching (square symbols) and/or by corresponding standards (circles). (**B**) Total Ion Chromatography (TIC) profile of cellular extract derived molecular features detected by GCxGC-MS. (**C**) 3-dimensional representation of GCxGC-MS analysis of a cellular extract, where GC (1st dimension) is on the X-axis, GC (2nd dimension) on the Y-axis and ion intensity is on the Z-axis.

**Fig. 2:**
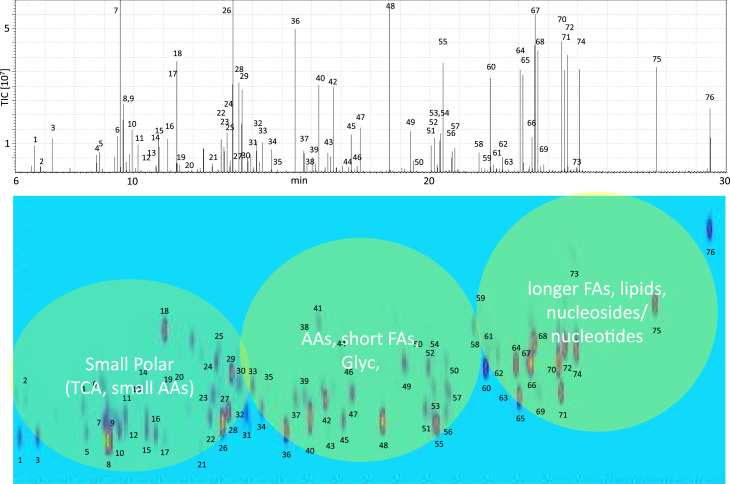
***2D map of standard metabolites***. 76 standard metabolites were analyzed by GCxGC-MS, demonstrating separation clusters of compound classes such as small polar metabolites, amino acids, fatty acids (Fas), glycolysis metabolites (Glyc.), longer fatty acids, lipids and nucleosides/nucleotides.

We first established a pipeline of metabolite extraction and chemical derivatization protocols optimized for blood (serum or plasma), urine, mammalian cells and tissue samples (Fig. S1). For mammalian cell extracts and homogenized tissue material, a methanol/water/tert-butyl methyl ether extraction was performed, followed by collecting the organic fraction and a subsequent second fractionation using excess methanol to isolate more polar compounds. As an alternative, the traditional Folch extraction (methanol/chloroform) was used, but no notable differences in terms of metabolite identification and quantitation were observed between the two methods, consistent with previous observations [32]. Also, no apparent changes were found when ratios of methanol/water and the order of extraction was altered. The methanol/water/tert-butyl methyl ether extraction was selected in our study due to its less toxic properties and easier handling. Both fractions were dried and subjected to chemical derivatization using methoxyamine and MSTFA, followed by immediate analysis using two-dimensional gas chromatography (GCxGC) using a combination of a non-polar and intermediate polarity column in the first and second dimension, respectively, coupled to a GP2010-single quadrupole mass spectrometer [16], [17]. The analysis of complex two-dimensional GC data has benefited from recent developments in high-speed acquisition MS technology, such as the GP2010 qMS system. Using this setup, we were able to detect 614 mass peaks (blobs) from a metabolic extract from the U2OS cell line, from which a considerable number were not separable by single-dimension GC-MS (Fig. 1A–C). A major challenge remains the accurate identification of such mass peaks despite the existence of comprehensive databases for electron impact (EI) spectra of standard metabolites and synthetic small molecules. Using the Chromsquare software matching algorithm, we identified 76 blobs/spots corresponding to standards (32 hits) or matching with metabolites in the NIST database (44 confident hits), thereby representing 12.4% of the molecular features detected (Fig. 1A). To further improve metabolite detection and confidence of identification, we prepared a “metabolite standard mix” of 76 compounds in a concentration range of 100 fmol–10 pmol/µl in order to create a GCxGC-qMS metabolite roadmap (Fig. 2, Table S2). Across the 2D-GC map, it is clearly possible to segregate metabolite classes based on their physico-chemical properties, where small polar molecules are eluting early, and lipophilic and nucleoside/nucleotides show longer retention times (Fig. 2). We used our standard panel to verify the confidence of metabolite identification by comparing EI spectral matches with biological sample derived metabolites versus corresponding standards. This allowed us to optimize matching parameters, in particular the similarity score that we found to be most optimal at 80 (100 max).

### 3.2 GCxGC boosts sensitivity and metabolite detection as compared to GC

**Fig. 3:**
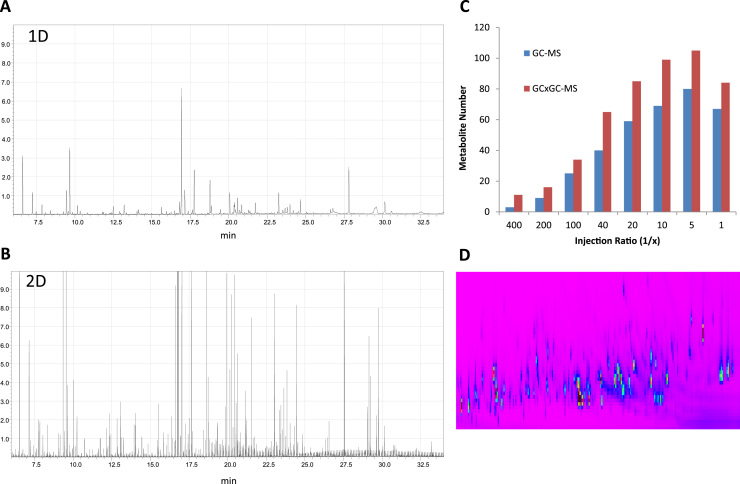
***GCxGC increases metabolite identification rate when compared to GC***. Metabolites extracted from a mouse liver were analyzed using 1D GC-qMS (**A**) or 2D GCxGC-MS (**B**). Injection of different amounts by varying the injection ratio (1/x) revealed a consistently higher number of detected and identified metabolites in GCxGC-MS (**C**), predominantly due to enhanced metabolite separation in the second dimension (**D**) that ameliorated identification. Metabolite detection numbers were similar in two technical duplicates that were analyzed, from which one is shown, and the metabolites identified as subsets of the list described in Table S2.

Clearly, increased peak and EI spectral intensities ameliorate metabolite identification, and we observed that 2D separation boosts sensitivity ~10- to 20-fold over 1D as demonstrated with two standards, methyl oleate and squalene, when analyzed by GC-qMS as compared to GCxGC-qMS (Fig. S2A, and B). This appears to be due to sample accumulation during modulation (6 s), which when released gave rise to much sharper peak shapes (Fig. S2, compare 1D versus 2D panels). As a consequence, cellular metabolites can be detected in the high-fmol to mid-pmol range on column (OC) as exemplified by determining the limit of detection (LOD) levels for adenosine (~15 fmol), cholesterol (~21 fmol), citric acid (19 fmol), creatinine (~70 fmol), glucose (~9 fmol), lactic acid (~94 fmol), myristic acid-14,14,14-d_3_ (~63 fmol), spermidine (~50 fmol) and tryptophan (~70 fmol) (Fig. S3A-H), thereby allowing detection of these metabolites at an endogenous level from a few million mammalian cells and from bio fluids in the µl range. The linear range for quantitation was between 0.025 and 100 pmol (OC), in which variabilities between 5.4–9.9% (CV) were achieved across multiple runs (Fig. S3). Combining the analysis of extracts prepared from cells, tissue, serum and urine, we were able to identify 165 distinct metabolic derivatives representing 155 unique metabolites (Table S3). The amino acids glycine, threonine, aspartic acid, methionine and tyrosine were observed as derivatives carrying multiple TMS groups. The extra dimension in gas chromatography based separation has been described to increase feature detection in serum and urine samples [33], [34]. To examine this in further detail, we explored the impact of multi-dimensional separation (GCxGC) on the identification of cellular metabolites as compared to GC. Variation of the modulation time for optimal separation on the first versus second dimension has been tested systematically [25]. Using a configuration of 6 s modulation time that balances separation between the two columns, we analyzed a mouse liver metabolite extract by 1D-GC versus 2D-GC, which revealed a more complex ion chromatogram pattern in the latter analysis (Fig. 3A and B), predominantly due to the concentration effect of cryo-modulation (Fig. S2). Consequently, we observed an increase in metabolite identification that pass the confidence threshold as a function of the amount of material analyzed by GC-qMS that was consistently elevated in GCxGC-qMS (Fig. 3C), in part due to enhanced separation of metabolic features in the second dimension (Fig. 3D).

### 3.3 Optimizing GCxGC-MS acquisition parameters

**Fig. 4:**
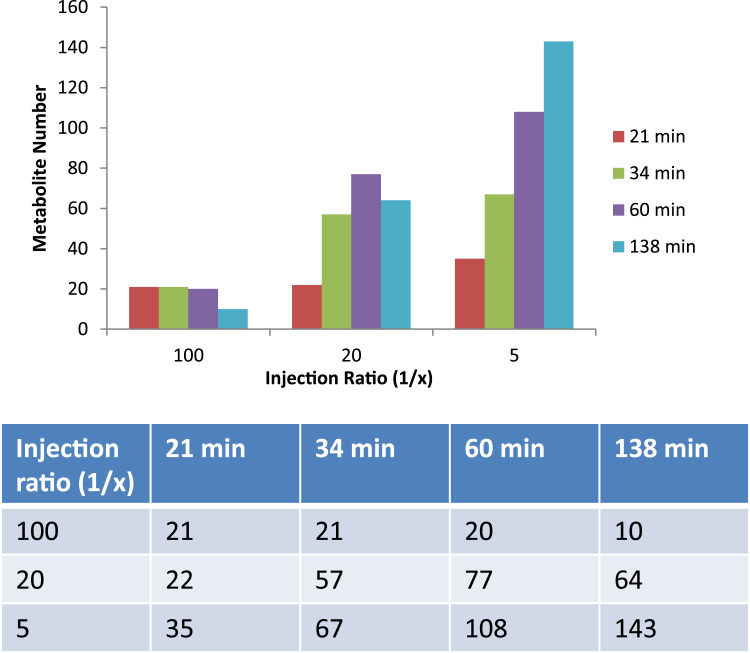
***Increased gradient length improves metabolite identification rates***. Metabolite extracts derived from MCF7 breast cancer cells were injected at different injection ratios (1/x) and separated using temperature slopes of either 20 °C/min, 10 °C/min, 5 °C/min or 2 °C/min between 60 and 320 °C that generated run lengths of 21, 34, 60 and 138 min (gradient +8 min at 320 °C), respectively. Longer gradients led to the identification of more metabolites, in particular when more material was injected. Metabolite detection numbers were similar in two technical duplicates that were analyzed, from which one is shown, and the metabolites identified as subsets of the list described in Table S2.

To further increase the number of metabolite identification, we also explored the impact of gradient length by varying temperature slopes. Using either 20 °C/min, 10 °C/min, 5 °C/min or 2 °C/min between 60–320 °C, we generated run lengths of 21, 34, 60 and 138 min (gradient +8 min at 320 °C), respectively (Fig. 4). We varied injection amounts of a metabolite extract derived from MCF7 cells by using different split ratios, and observed a clear trend of the number of metabolites identified as a function of gradient length and sample amount (Fig. 4). Interestingly, there appears to be an optimum in sample amount as this leads to detector saturation and subsequent changes in peak pattern ratios in EI spectra that impairs metabolite identification (not shown). Together, we conclude that longer gradients are beneficial for expanding metabolite identification numbers as more sample material can be injected, but at the cost of sample throughput.

### 3.4 Improved separation and differential chemical derivatization improves metabolite ID

**Fig. 5:**
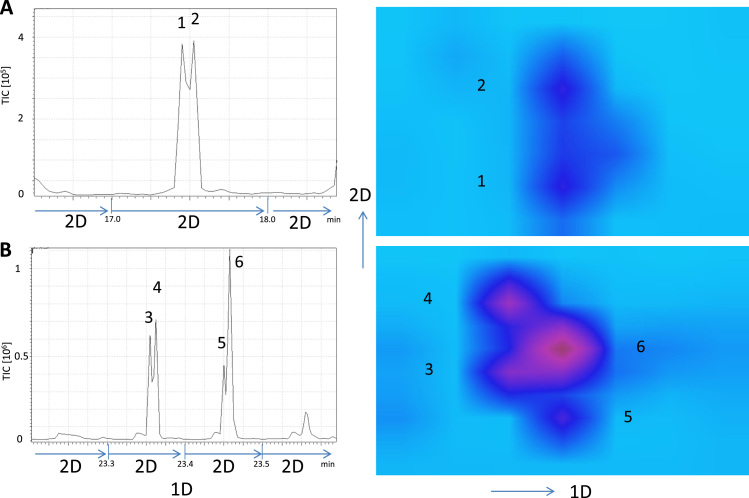
***GCxGC-MS improves separation of distinct metabolite species***. (**A**) D-talopyranose (**1**) and methyl palmitate (**2**) can be separated by a 6 s orthogonal GC separation. (**B**) Improved separation of inosine (**3**), diisooctyl phalate (DiOP, a plasticizer contaminant (**4**), monopalmitin (**5**) and docosahexaenoic acid (DHA, **6**) by GCxGC such that their IM spectra yielded confident identification.

**Fig. 6:**
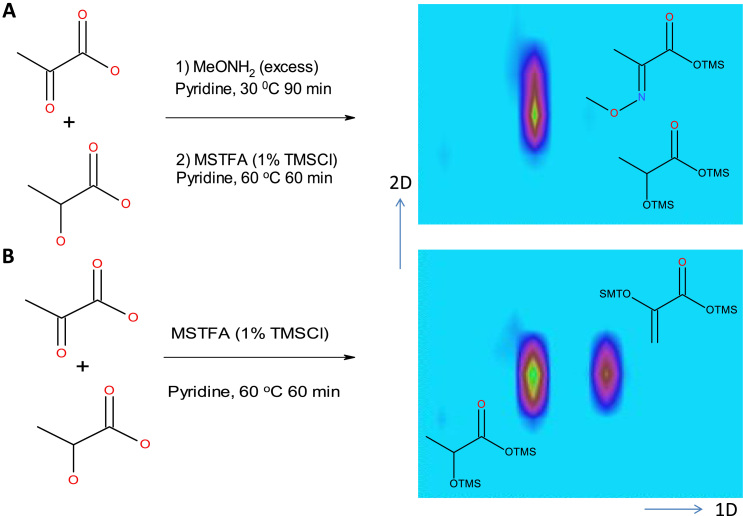
***Differential chemical derivatization allows efficient separation of lactate and pyruvate.*** (**A**) Chemical derivatization of lactate and pyruvate containing samples using MeONH_2_ and subsequently MSTFA (1% TMSCI) yielded in derivatization products that were not separable by GCxGC-MS. (**B**) A one step derivatization protocol using MSTFA (1% TMSCI) improved separation of lactate and pyruvate.

Closer examination of 2D GC maps revealed that metabolites of different categories appear to co-migrate on single-dimension GC that can be clearly separated by GCxGC. For instance, D-talopyranose (**1**) and methyl palmitate (**2**) can be separated by a 6 s orthogonal GC separation (Fig. 5A), and the EI spectra of the separated compounds were of sufficient quality for confident identification (Fig. S4 A–C). In a similar fashion, we observed separation of inosine (**3**), diisooctyl phalate (DIOP (**4)**, a plasticizer contaminant, monopalmitin (**5**) and docosahexaenoic acid (DHA, **6**) by GCxGC such that their IM spectra yielded confident identification (Fig. 5B and S4 D–H). In other cases, such as lactate and pyruvate, GCxGC was unable to provide sufficient resolution for adequate separation as the corresponding derivatization products co-migrated in both dimensions (Fig. 6A). Interestingly, when we omitted the first chemical derivatization step using methoxyamine (MeONH_2_) and only used MSTFA and TMSCI, the trimethylsilane adducts of lactate and pyruvate were sufficiently different to be separable (Fig. 6B), providing evidence that variations in chemical modification strategies may further increase molecular feature detection in complex biomedical samples.

## 4 Conclusions

In this report, we optimized a two-dimensional gas chromatography separation coupled to fast-scanning mass spectrometry for the profiling of a panel of 165 metabolite derivatives/155 unique metabolites present in clinical samples. Robust metabolite extraction and chemical derivatization protocols were established for mammalian cells, tissue material, urine and serum/plasma. Identification of metabolites is based on the comparison to standards and database matching, and linear quantitation observed in the fmol to pmol range. We show that GCxGC-qMS has increased sensitivity over GC-qMS and that two-dimensional separation as well as extended gradients increase the panel of identifiable metabolites. Our findings extend the utility of GCxGC-qMS as a useful platform that is complementary to existing analytical methodologies in biomedical metabolomics.

## Declaration

The authors declare no competing financial interests in the context of the work described in this manuscript.

## Appendix A Supplementary material

Figure S1 ***Metabolite extraction and chemical derivatization workflows for samples derived from the clinic***. These include heart, kidney or brain tissue from pig (5mg wet tissue weight), cellular material (typically 5×10^6^ cells, in this study derived from U2OS/T24 cell lines), serum/plasma (pig) and urine (pig). Tissue and cellular material is first homogenized prior to extraction, and liquid samples directly subjected to metabolite extraction using tert-butyl methyl ester. The organic layer is harvested and the remaining material is subjected to a second extraction step using methanol. Both liquid layers are dried, subjected to chemical derivatization using methoxyamine and MSTFA (1% TMSCI) and directly analyzed by GC×GC-MS. Data analysis includes processing using GC-Solution (acquisition), GC-Image (feature matching using NIST database) and Chromsquare (quantitation) software.

.

Figure S2 ***Increased sensitivity of 2D versus 1D GC*.** The standards methyl oleate (**A**) and squalene (**B**) were used to compare sensitivity in GC-qMS versus GC×GC-qMS mode. Standards were injected at concentrations between 250fmol and 25pmol and analyzed using GC-qMS (1D) or GC×GC-qMS (2D) on a 34 min gradient. Peaks occurring on a regular pattern with 6 s intervals are background, and signals of standards are indicated with an arrow. Inserts show condensed views of 1D chromatograms.

.

Figure S3 ***Dynamic range of metabolite detection by GC***×***GC-MS***. Quantitative measurement of a selected panel of metabolites analyzed by GC×GC-MS in the range of 10fmol to 100pmol. (**A**) Adenosine; (**B**) Cholesterol; (**C**) Citric acid; (**D**) Creatinine; (**E**) Glucose; (**F**) Lactic acid; (**G**) [^2^H]- Myristic acid (methyl-D3); (**H**) Spermidine and (**I**) Tryptophan. Coefficient of variation (CV), limit of detection (L.O.D.) and the linear range were assessed across four injection repeats.

.

Figure S4 ***Electron impact (EI) spectra of metabolite species identified in Figure 7***. (**A**) EI spectrum of unresolved spots (blobs) containing D-(+)-talopyranose (1) and methyl palmitate (2). (**B**) EI spectrum of D-(+)-talopyranose (1). (**C**) EI spectrum of methyl palmitate (2). (**D**) EI spectrum of unresolved spots (blobs) containing inosine (3), DIOP (4), monopalmitin (5) and 4,7,10,13,16,19-Docosahexaenoic acid (6). (**E**) EI spectrum of inosine (3). (**F**) EI spectrum of DIOP (4). (**G**) EI spectrum of monopalmitin (5). (**H**) EI spectrum of 4,7,10,13,16,19-Docosahexaenoic acid (6). See also Figure 6 for additional details.

.

Table S1 Optimized parameters for GC×GC-MS data analysis.

.

Table S2 Metabolite standards separated by GC×GC-MS.

.

Table S3 Metabolites identified by GC×GC-MS in cell, tissue, plasma and urine.

## Footnotes

Appendix A Supplementary data associated with this article can be found in the online version at doi:10.1016/j.talanta.2017.01.003.
