# Supplementary figures and images for: Optimizing 2D gas chromatography mass spectrometry for robust tissue, serum and urine metabolite profiling
Source: Talanta. 2017 Apr 1;165:685–91. doi: 10.1016/j.talanta.2017.01.003 (PMC5294743; doi:10.1016/j.talanta.2017.01.003)

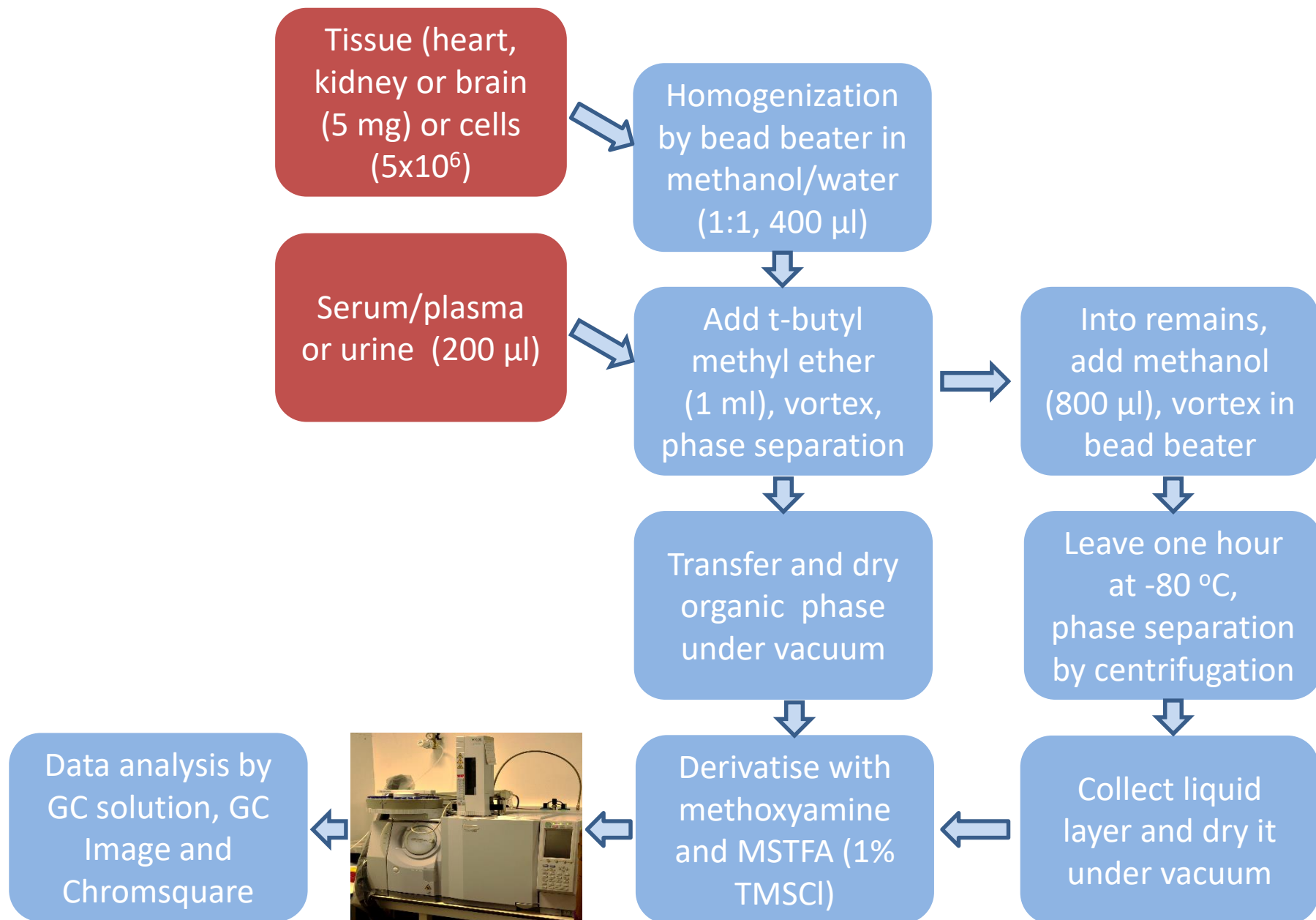

Analysis by GCxGC-MS

Figure S1

Supplement: Supplementary file 1 — Figure S1 Metabolite extraction and chemical derivatization workflows for samples derived from the clinic. These include heart, kidney or brain tissue from pig (5mg wet tissue weight), cellular material (typically 5×10^6^ cells, in this study derived from U2OS/T24 cell lines), serum/plasma (pig) and urine (pig). Tissue and cellular material is first homogenized prior to extraction, and liquid samples directly subjected to metabolite extraction using tert-butyl methyl ester. The organic layer is harvested and the remaining material is subjected to a second extraction step using methanol. Both liquid layers are dried, subjected to chemical derivatization using methoxyamine and MSTFA (1% TMSCI) and directly analyzed by GC×GC-MS. Data analysis includes processing using GC-Solution (acquisition), GC-Image (feature matching using NIST database) and Chromsquare (quantitation) software. [file mmc1.pdf]

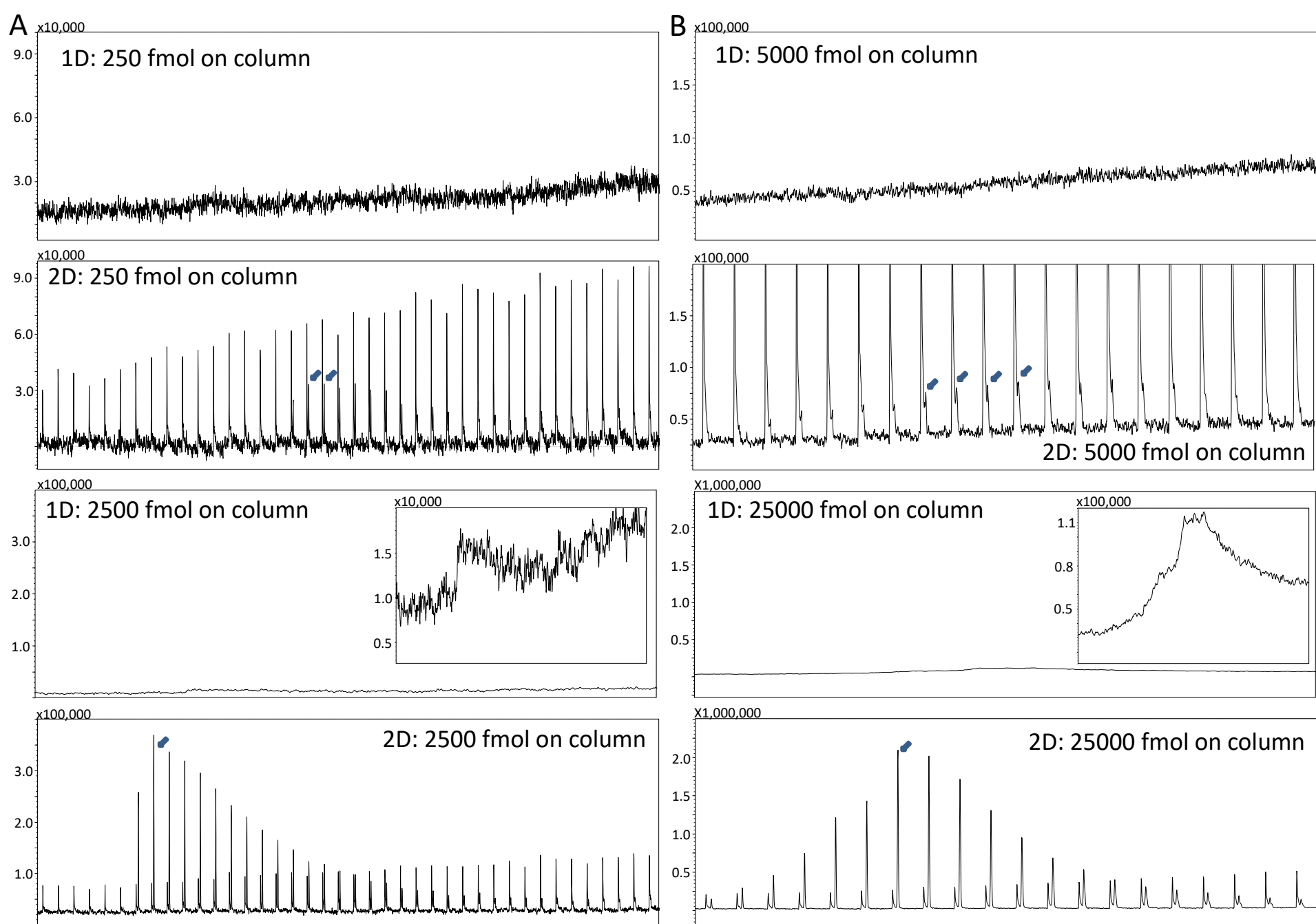

Figure S2

Supplement: Supplementary file 2 — Figure S2 Increased sensitivity of 2D versus 1D GC. The standards methyl oleate (A) and squalene (B) were used to compare sensitivity in GC-qMS versus GC×GC-qMS mode. Standards were injected at concentrations between 250fmol and 25pmol and analyzed using GC-qMS (1D) or GC×GC-qMS (2D) on a 34 min gradient. Peaks occurring on a regular pattern with 6 s intervals are background, and signals of standards are indicated with an arrow. Inserts show condensed views of 1D chromatograms. [file mmc2.pdf]

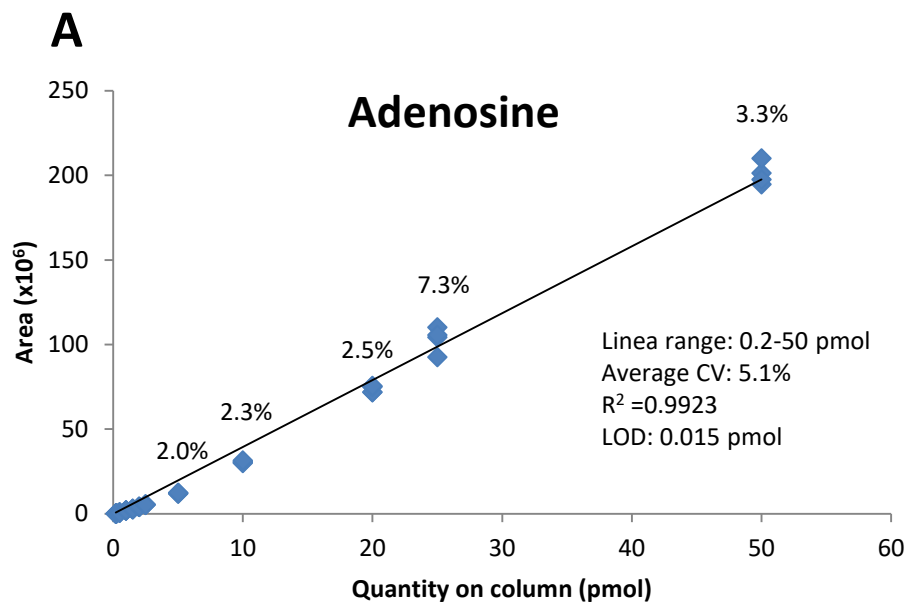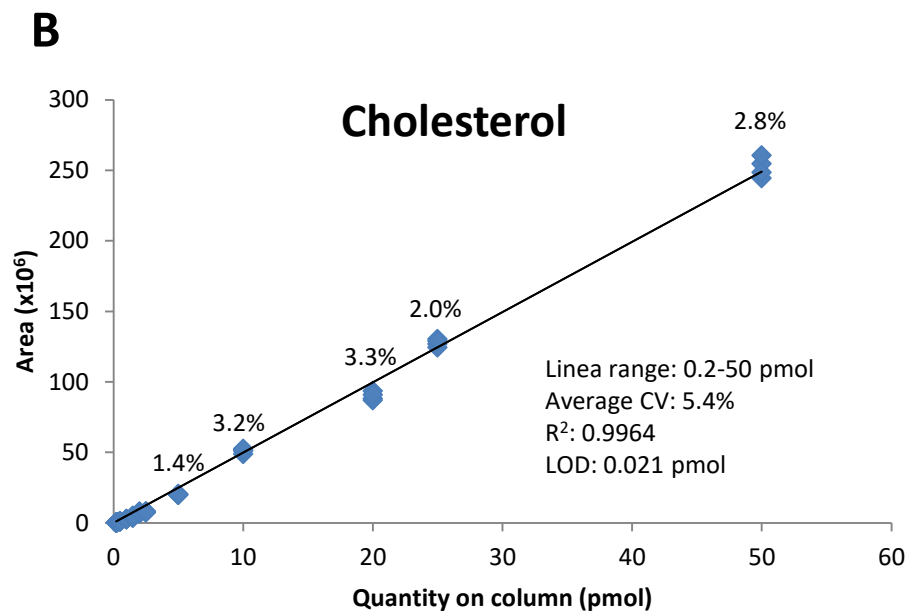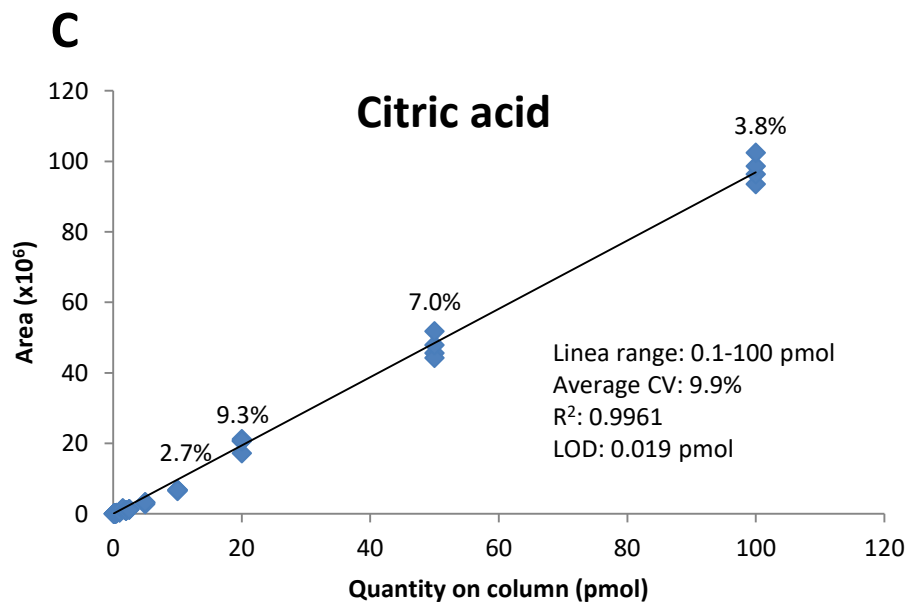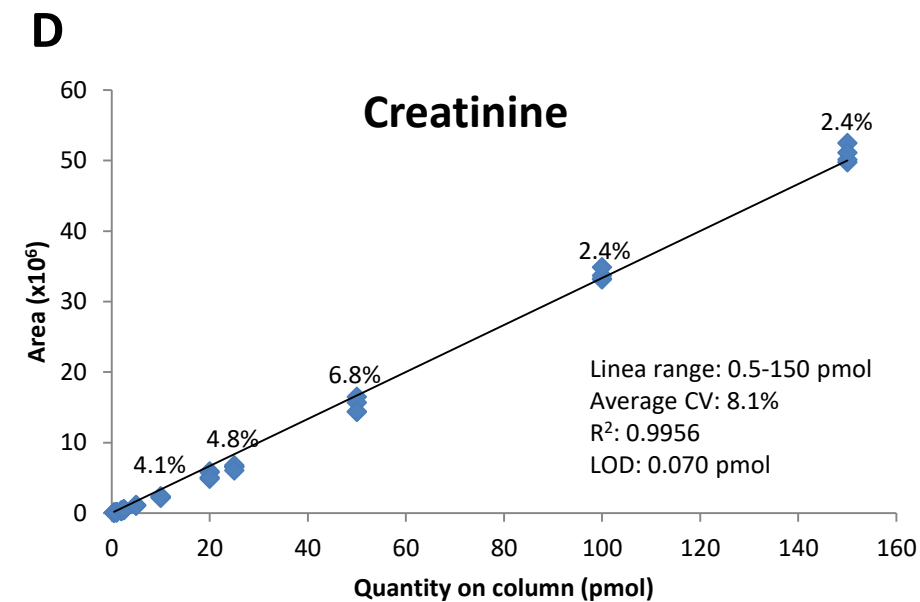

Figure S3

**E**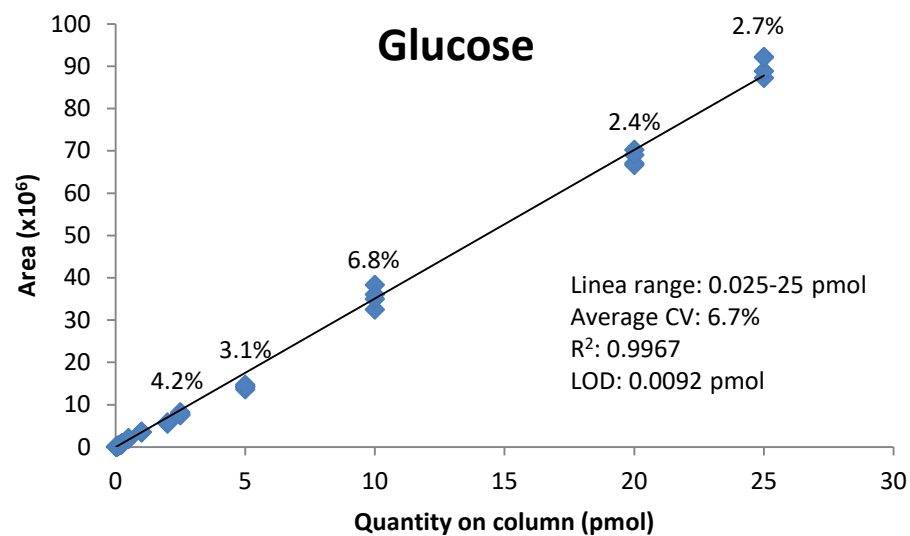**F**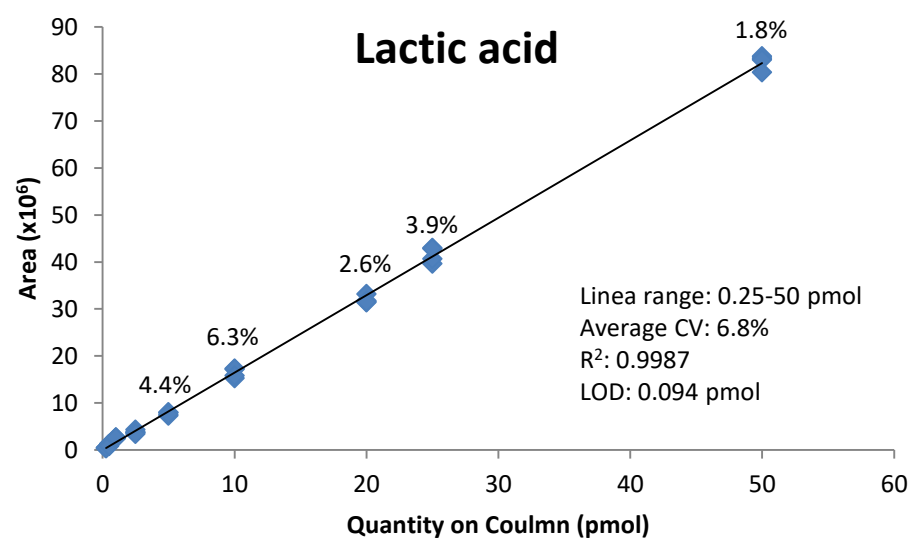**G**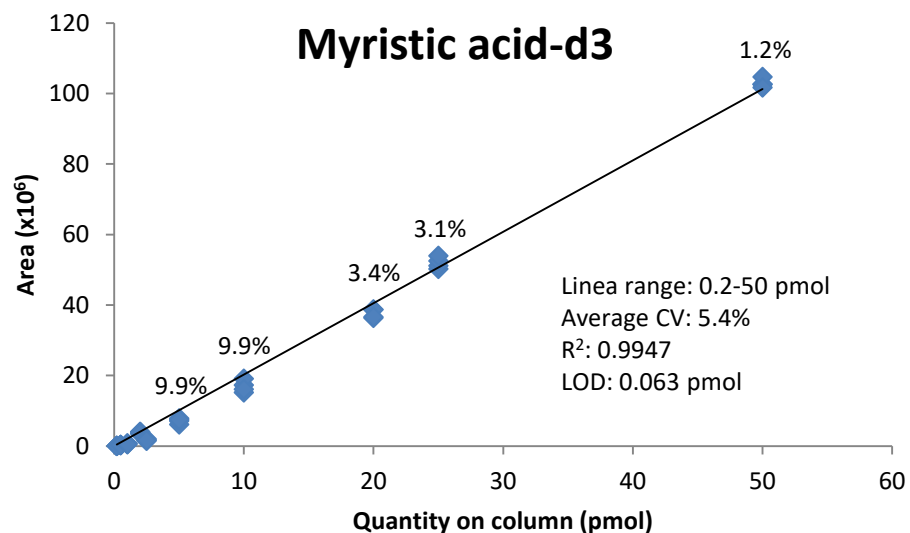**H**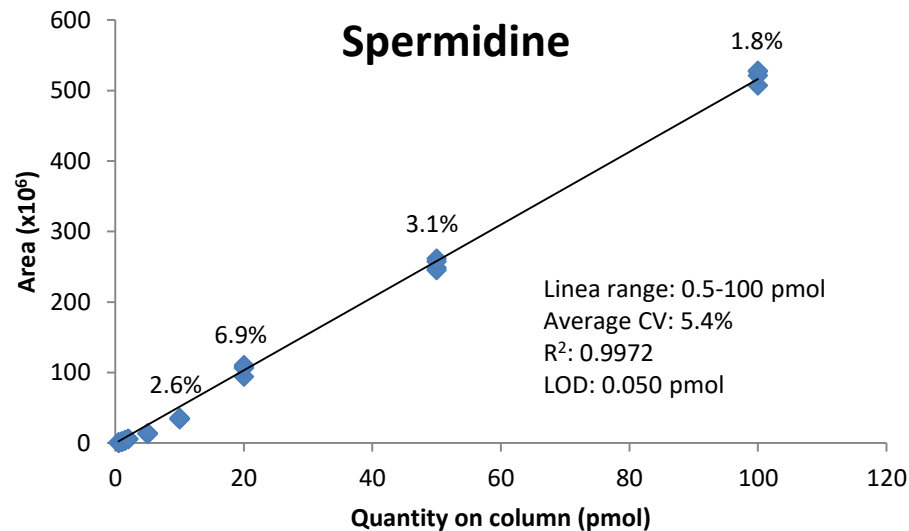

Figure S3

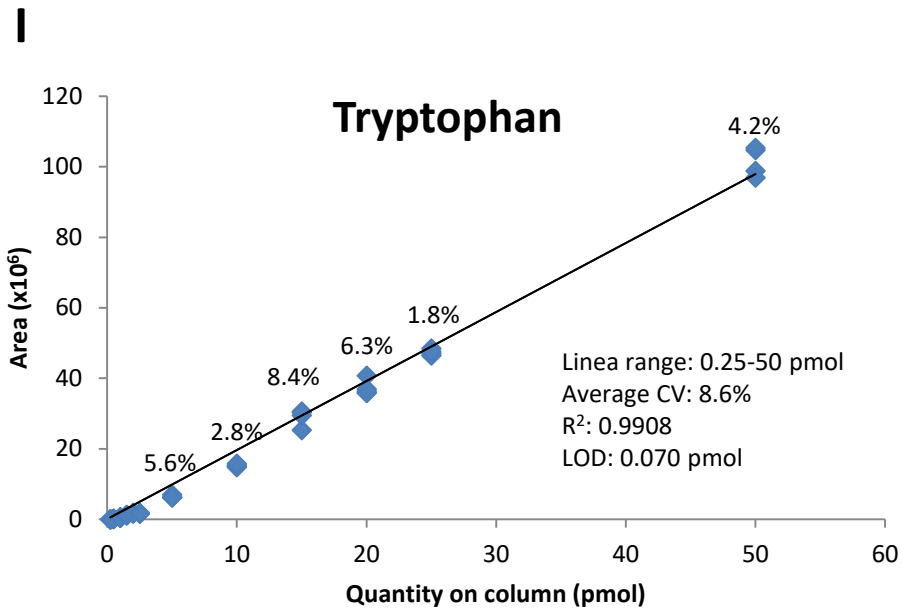

Figure S3

Supplement: Supplementary file 3 — Figure S3 Dynamic range of metabolite detection by GC×GC-MS. Quantitative measurement of a selected panel of metabolites analyzed by GC×GC-MS in the range of 10fmol to 100pmol. (A) Adenosine; (B) Cholesterol; (C) Citric acid; (D) Creatinine; (E) Glucose; (F) Lactic acid; (G) [2H]- Myristic acid (methyl-D3); (H) Spermidine and (I) Tryptophan. Coefficient of variation (CV), limit of detection (L.O.D.) and the linear range were assessed across four injection repeats. [file mmc3.pdf]
