# Supplementary material for: Optimizing 2D gas chromatography mass spectrometry for robust tissue, serum and urine metabolite profiling
Source: Talanta. 2017 Apr 1;165:685–91. doi: 10.1016/j.talanta.2017.01.003 (PMC5294743; doi:10.1016/j.talanta.2017.01.003)
Supplement: Supplementary file 4 — Figure S4 Electron impact (EI) spectra of metabolite species identified in Figure 7. (A) EI spectrum of unresolved spots (blobs) containing D-(+)-talopyranose (1) and methyl palmitate (2). (B) EI spectrum of D-(+)-talopyranose (1). (C) EI spectrum of methyl palmitate (2). (D) EI spectrum of unresolved spots (blobs) containing inosine (3), DIOP (4), monopalmitin (5) and 4,7,10,13,16,19-Docosahexaenoic acid (6). (E) EI spectrum of inosine (3). (F) EI spectrum of DIOP (4). (G) EI spectrum of monopalmitin (5). (H) EI spectrum of 4,7,10,13,16,19-Docosahexaenoic acid (6). See also Figure 6 for additional details. [file mmc4.pdf]

A

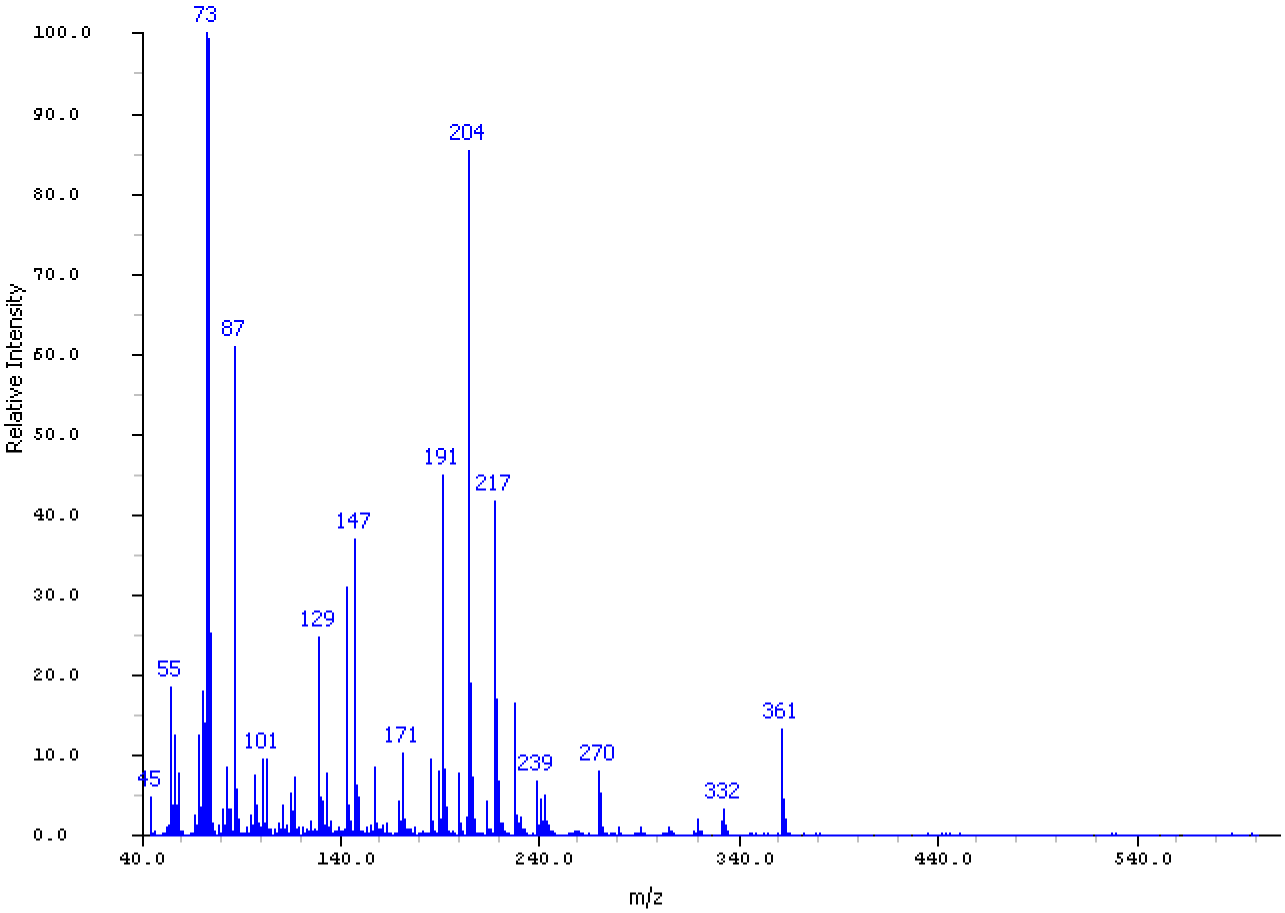

73
204
87
191
217
147
129
55
101
171
361
45
239
270
332
100.0
90.0
80.0
70.0
60.0
50.0
40.0
30.0
20.0
10.0
0.0
Relative Intensity
40.0
140.0
240.0
340.0
440.0
540.0
m/z

Figure S4

B

D-(+)-Talopyranose (1)

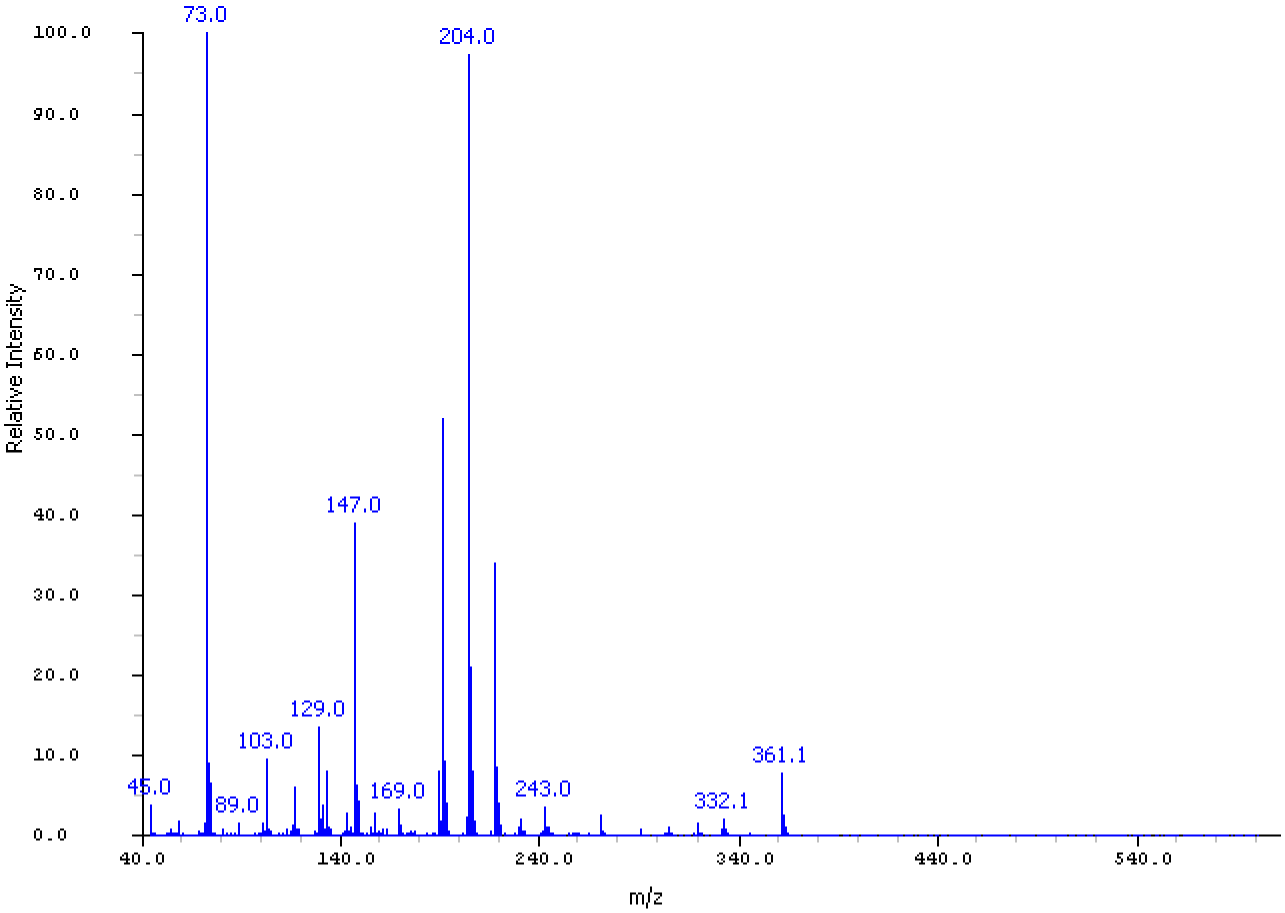

Figure S4

C

Methyl Palmitate (2)

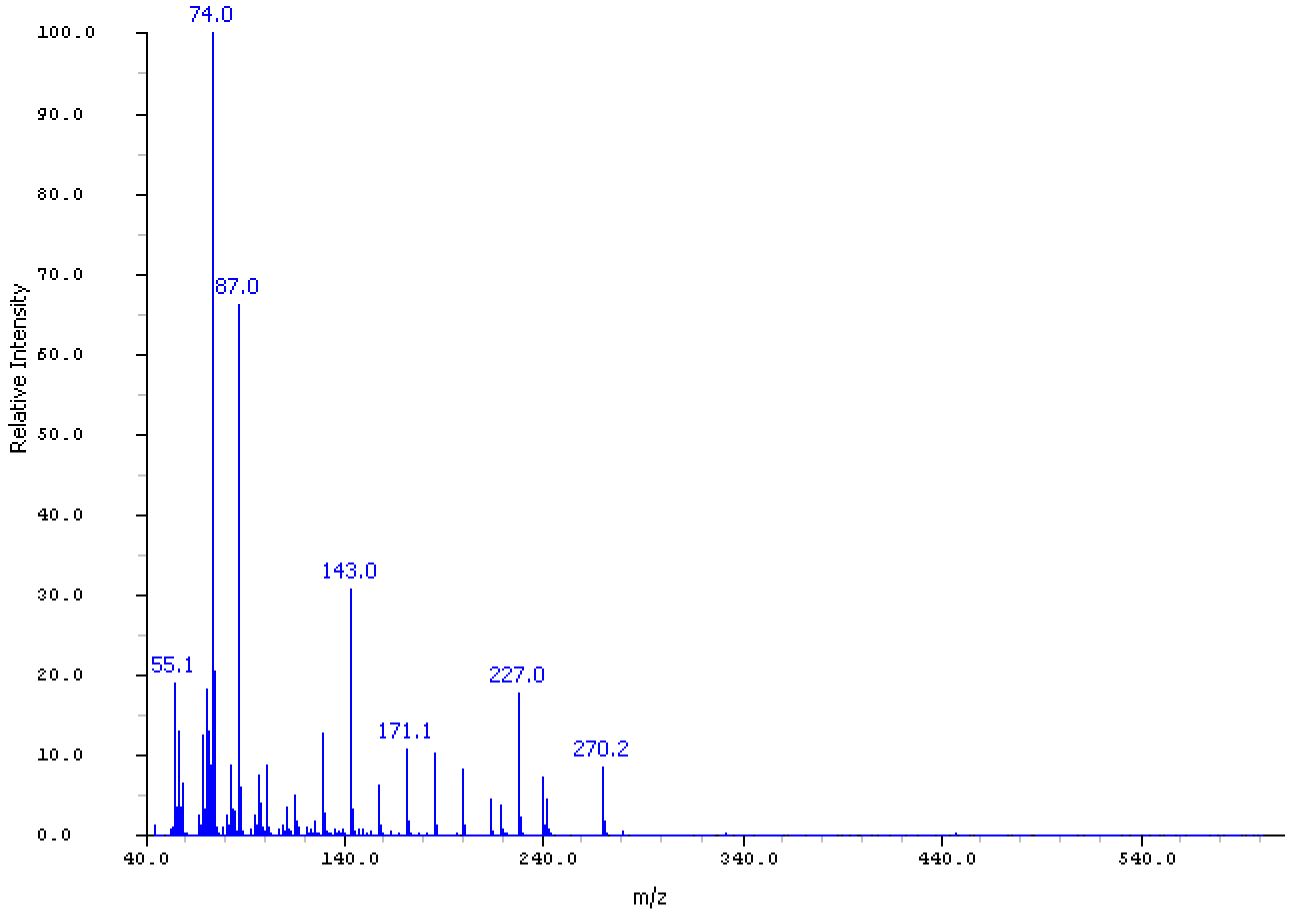

Figure S4

D

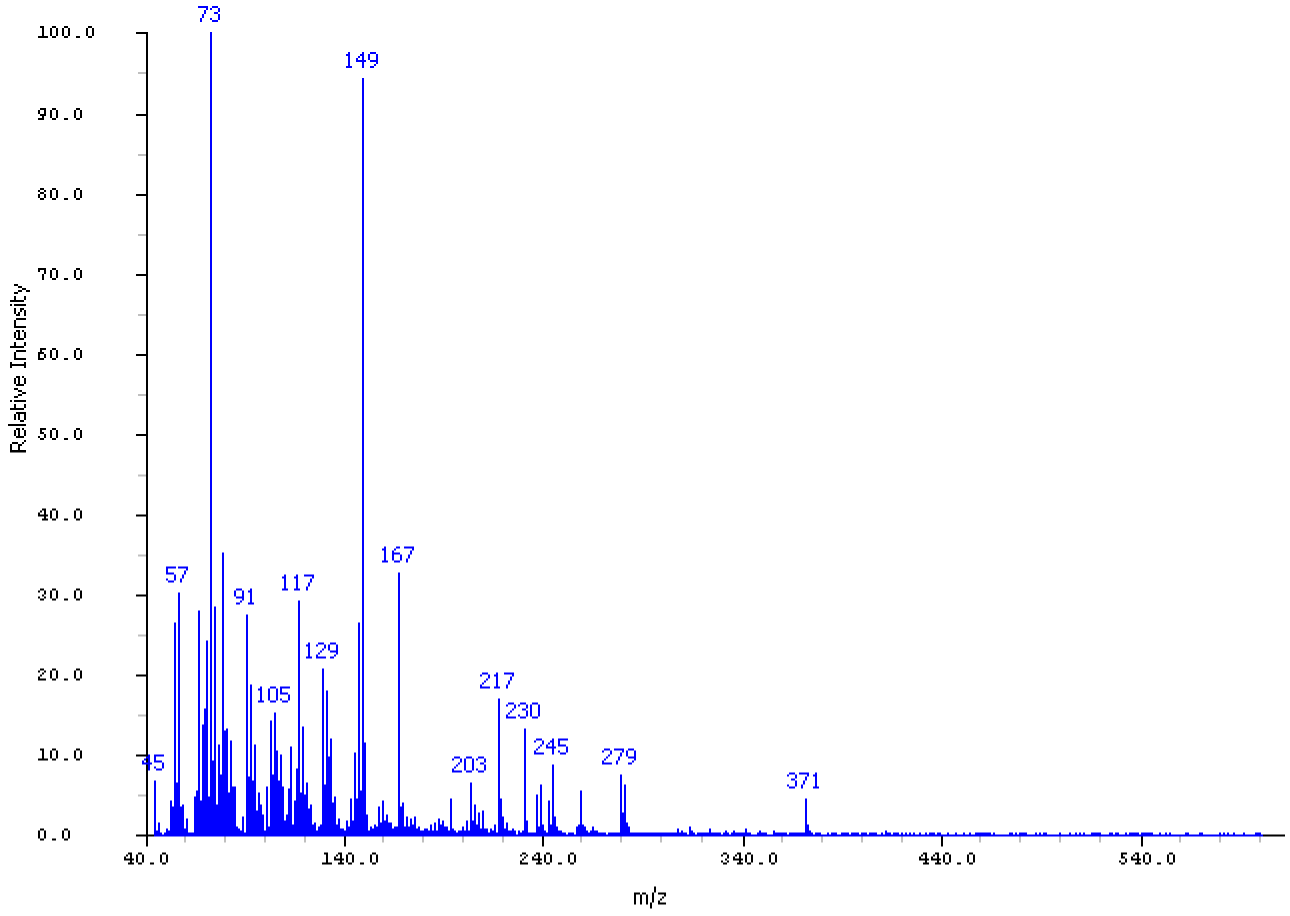

73
149
167
57
91
117
129
105
217
230
245
279
203
45
371
100.0
90.0
80.0
70.0
60.0
50.0
40.0
30.0
20.0
10.0
0.0
Relative Intensity
40.0
140.0
240.0
340.0
440.0
540.0
m/z

Figure S4

E

Inosine (3)

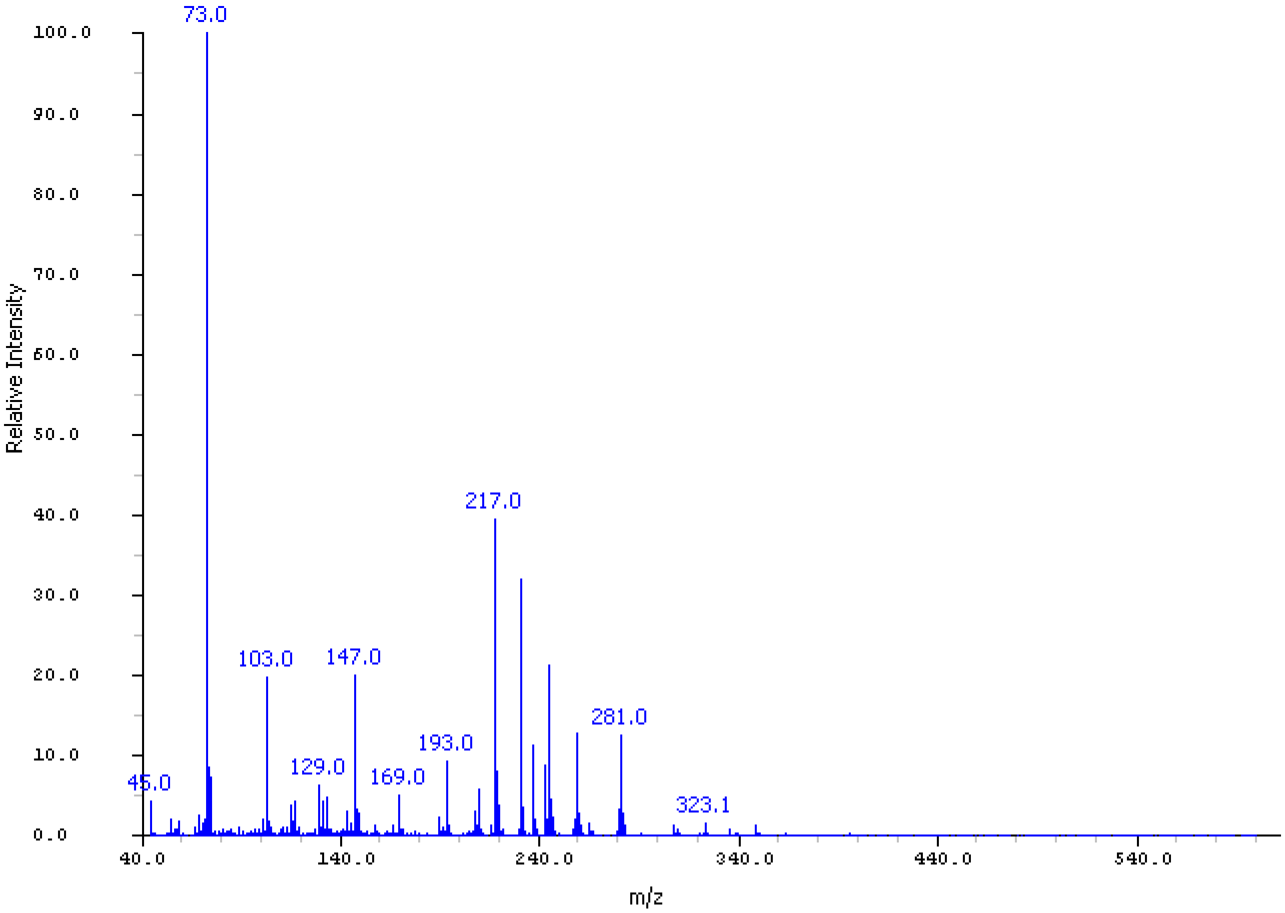

Figure S4

F

DIOP (4)

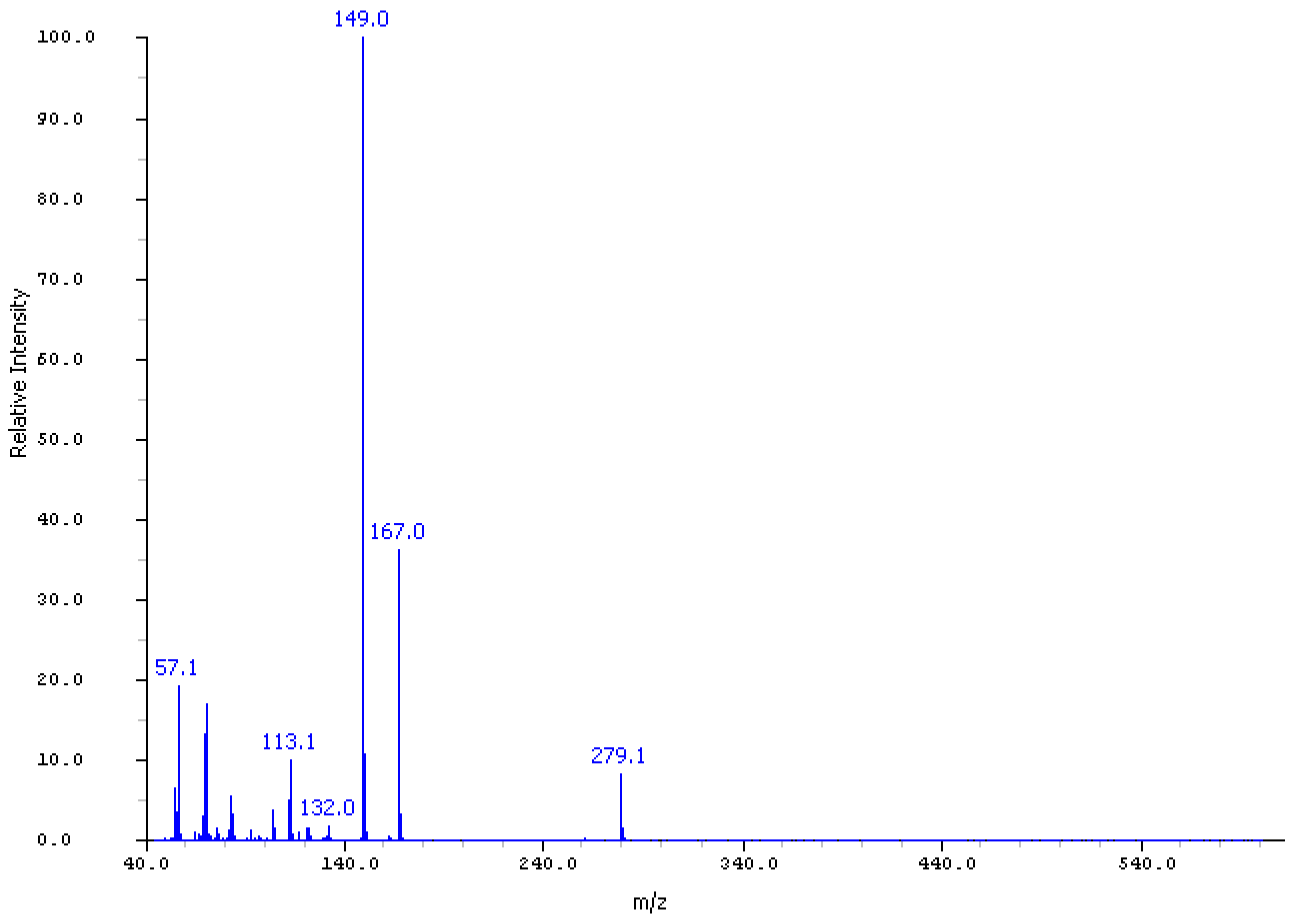

Figure S4

G

Monopalmitin (5)

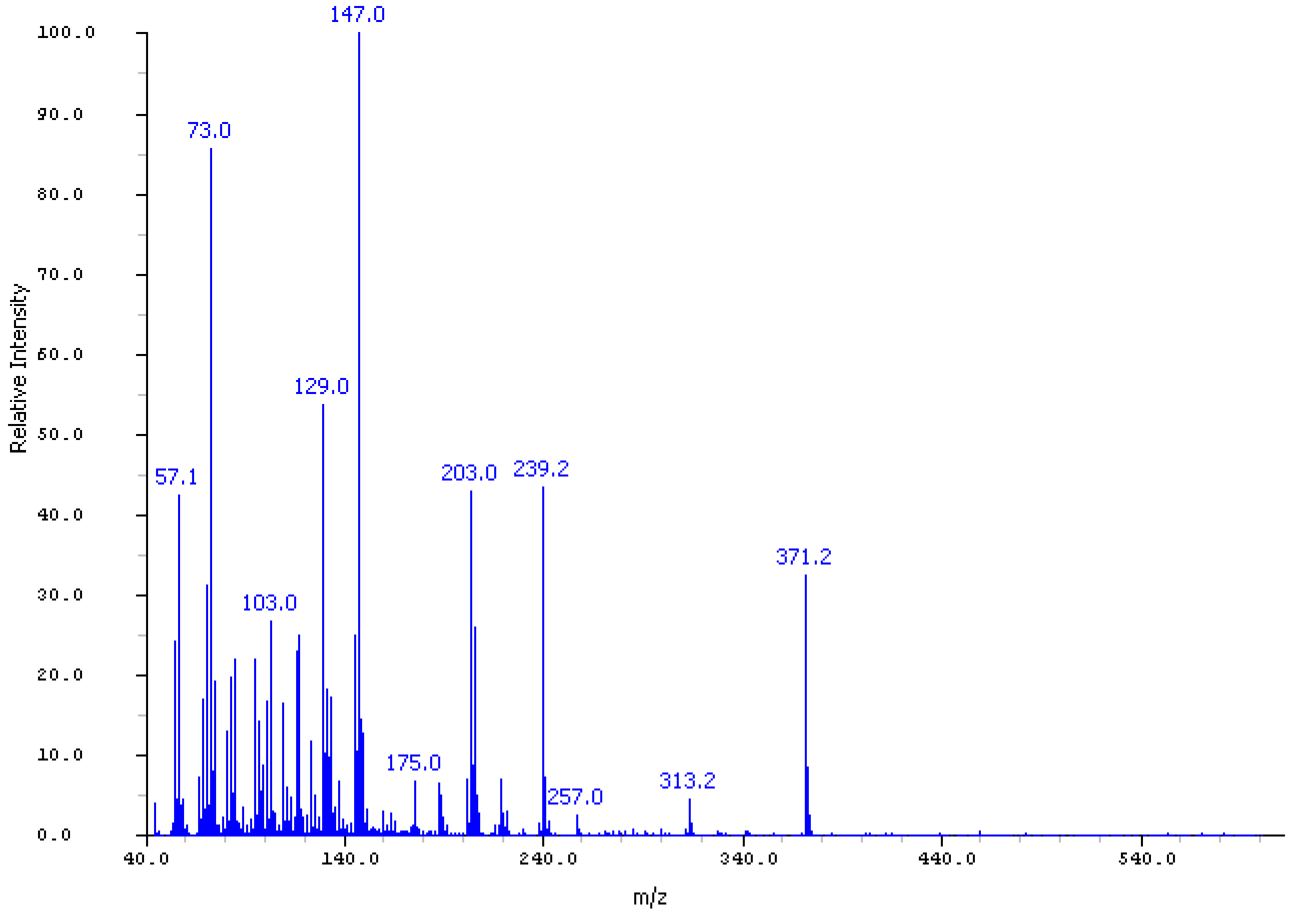

Figure S4

H 4,7,10,13,16,19-Docosahexaenoic acid (6)

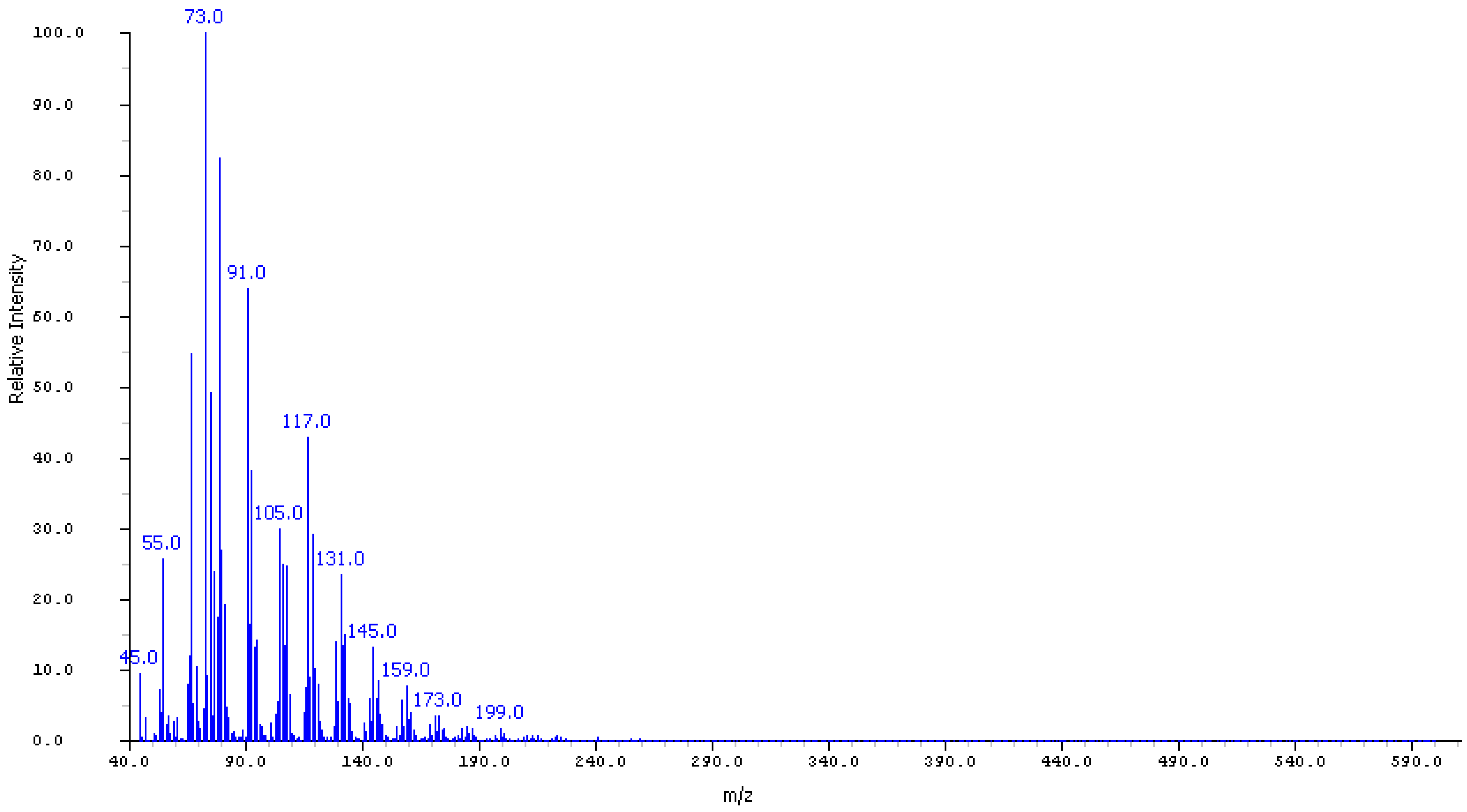

Figure S4
