# Supplementary material for: Optimizing 2D gas chromatography mass spectrometry for robust tissue, serum and urine metabolite profiling
Source: Talanta. 2017 Apr 1;165:685–91. doi: 10.1016/j.talanta.2017.01.003 (PMC5294743; doi:10.1016/j.talanta.2017.01.003)
Supplement: Supplementary file 5 — Table S1 Optimized parameters for GC×GC-MS data analysis. [file mmc5.pdf]

Table S1. Optimized parameters for GCxGC-MS data analysis

Injection [µl]	Ratio [1/x]	Slope [min^{-1}]	Reference
0.5	200	7200	Our study
1	200	22000	Our study
1	100	22000	Our study
1	40	22000	Our study
1	20	22000	Our study
1	10	68000	Our study
1	5	230000	Our study
1	1	440000	Our study
-	10	5000	17
