# Supplementary material for: Optimizing 2D gas chromatography mass spectrometry for robust tissue, serum and urine metabolite profiling
Source: Talanta. 2017 Apr 1;165:685–91. doi: 10.1016/j.talanta.2017.01.003 (PMC5294743; doi:10.1016/j.talanta.2017.01.003)
Supplement: Supplementary file 6 — Table S2 Metabolite standards separated by GC×GC-MS. [file mmc6.pdf]

Table S1: Standard metabolites and their retention time

ID	Compound Name	t^{1D}_R (min)	t^{2D}_R (sec)
1	Lactic acid-2TMS	6.641	2.4
2	Glycolic acid-2TMS	6.843	2.5
3	L-Alanine-2TMS	7.241	2.45
4	Malonic acid-2TMS	8.745	2.65
5	L-Valine-2TMS	8.842	2.45
6	Urea-2TMS	9.348	2.85
7	Glycerol-3TMS	9.54	2.35
8	L-Leucine-2TMS	9.64	2.35
9	Phosphoric acid-3TMS	9.644	2.6
10	L-Isoleucine-2TMS	9.942	2.45
11	L-Proline-2TMS	10.144	2.6
12	Glyceric acid-3TMS	10.442	2.45
13	β-Hydroxypyruvic acid-2TMS	10.546	2.7
14	Pyrimidine-2TMS	10.748	2.8
15	L-Serine-3TMS/Fumaric acid-2TMS	10.842	2.5
16	L-Threonine-3TMS	11.142	2.5
17	Tris-3TMS	11.441	2.45
18	Mevalonic lactone-TMS	11.455	3.3
19	Thymine-2TMS	11.548	2.85
20	L-Aspartic acid-2TMS	11.848	2.85
21	Tris-4TMS	12.64	2.4
22	L-Aspartic acid-3TMS	12.943	2.6
23	L-Methionine-2TMS	13.047	2.8
24	5-Oxoproline-2TMS	13.151	3.05
25	Cytosine-2TMS	13.253	3.15
26	Mevalonic acid-3TMS	13.343	2.55
27	Creatinine enol-3TMS	13.446	2.8
28	2-hydroxyglutaric acid-3TMS	13.544	2.65
29	2-Ketoglutaric acid-methyloxime-2TMS	13.649	2.95
30	Phosphoenol pyruvic acid-3TMS	13.848	2.9
31	L-Ornithine-3TMS	14.043	2.55
32	L-Glutamic acid-3TMS	14.144	2.65
33	L-Phenylalnine-2TMS	14.348	2.85
34	Homocysteine-3TMS	14.645	2.7
35	Taurine-3TMS	14.848	2.85
36	Putrescine-4TMS	15.442	2.5
37	2-Phosphoglyceric acid-3TMS	15.746	2.75
38	L-Glutamine-3TMS	15.85	2.8
39	3-Phosphoglyceric acid-3TMS	16.047	2.8
40	Citric acid-4TMS	16.244	2.65
41	9H-Purine-2TMS	16.556	3.35
42	Myristic acid-d3-TMS	16.746	2.75
43	D-Glucose-methyloxime-5TMS	16.842	2.5
44	L-Tyrosine-2TMS	17.252	3.1
45	L-Lysine-4TMS	17.343	2.6
46	L-Histidine-3TMS	17.451	3.05
47	L-Tyrosine-3TMS	17.647	2.8

48	1,7-Diaminoheptane-4TMS	18.643	2.6
49	Methyl oleate	19.351	3.05
50	9H-Purin-2-amine-3TMS	19.555	3.3
51	Spermidine-4TMS	20.045	2.7
52	11-cis-Vaccenyl acetate	20.15	3
53	Oleic acid-TMS	20.348	2.85
54	L-Tryptophan-3TMS	20.352	3.1
55	Spermidine-5TMS	20.443	2.55
56	D-Glucose-6-phosphate-6TMS	20.744	2.65
57	L-Cystine-4TMS	20.847	2.8
58	Deoxyuridine-2TMS	21.656	3.35
59	Oleamide	21.858	3.5
60	Oleamide-TMS	22.05	3
61	Deoxyuridine-3TMS	22.153	3.15
62	Thymidine-3TMS	22.452	3.1
63	Homocystine	22.647	2.85
64	Inosine-4TMS	23.051	3.05
65	Monopalmitin-2TMS	23.146	2.75
66	5-Methyluridine-3TMS	23.451	3.05
67	Adenosine-4TMS	23.551	3.05
68	Deoxyadenosine-3-TMS	23.653	3.2
69	Spermine-6TMS	23.847	2.8
70	Guanosine-5TMS	24.451	3.05
71	Monostearin-2TMS	24.547	2.8
72	2-Deoxyguanosine-4TMS	24.653	3.2
73	Cytidine-3TMS	24.965	3.9
74	Squalene/N-Oleoyl-Glycine-TMS	25.053	3.15
75	Cholesterol-TMS	27.658	3.45
76	Lanosterol-TMS	29.468	4.05
