# Supplementary material for: Optimizing 2D gas chromatography mass spectrometry for robust tissue, serum and urine metabolite profiling
Source: Talanta. 2017 Apr 1;165:685–91. doi: 10.1016/j.talanta.2017.01.003 (PMC5294743; doi:10.1016/j.talanta.2017.01.003)
Supplement: Supplementary file 7 — Table S3 Metabolites identified by GC×GC-MS in cell, tissue, plasma and urine. [file mmc7.pdf]

Table 2: Metabolites identified manually by similarity search in cell, tissue, plasma and urine samples

ID	Compound Name	t^{1D}_R (min)	t^{2D}_R (sec)
1	N,N-Dimethylglycine-1TMS	6.142	2.45
2	Lactic acid-2TMS	6.541	2.4
3	Alanine-2TMS	7.241	2.45
4	2-Hydroxyisobutyric acid-2TMS	7.542	2.45
5	3-Hydroxybutyric acid-2TMS	8.042	2.45
6	Oxalic acid-2TMS	8.048	2.7
7	2-Aminobutyric acid-2TMS	8.142	2.45
8	Leucine-TMS	8.145	2.65
9	Monomethyl phosphoric acid-2TMS	8.347	2.8
10	Proline-TMS	8.548	2.85
11	Valine-2TMS	8.842	2.45
12	2-Ketoisocaproic acid-Methyloxime-TMS	8.847	2.75
13	Ethanolamine-3TMS	9.041	2.4
14	*N*-Ethyl-hexahydro-1H-azepine	9.36	3.55
15	Urea-2TMS	9.448	2.8
16	Glycerol-3TMS	9.54	2.35
17	Serine-2TMS	9.545	2.70
18	Phosphoric acid-3TMS	9.644	2.6
19	Benzoic acid-TMS	9.65	2.95
20	Isoleucine-2TMS	9.942	2.45
21	Threonine-2TMS	10.044	2.6
22	Glycine-3TMS	10.142	2.45
23	Butanedioic acid-2TMS	10.345	2.65
24	Glyceric acid-3TMS	10.442	2.45
25	Pyrimidine-2TMS	10.648	2.85
26	2-Butenedioic acid (E)-2TMS	10.744	2.55
27	Picolinic acid-TMS	10.758	3.45
28	Serine-3TMS	10.842	2.5
29	Pyrazine-2TMS	10.844	2.65
30	Nonanoic acid-TMS	11.048	2.85
31	Threonine-3TMS	11.141	2.45
32	Tris-3TMS	11.341	2.45
33	β-Alanine-3TMS	11.743	2.55
34	Aspartic acid-2TMS	11.847	2.8
35	Methionine-TMS	11.853	3.15
36	Malic acid-3TMS	12.443	2.6
37	*meso*-Erythritol-4TMS	12.54	2.4
38	Aspartic acid-3TMS	12.844	2.65
39	Erythronic acid-4TMS	12.943	2.7
40	Niacinamide-TMS	12.858	3.45
41	4-Aminobutyric acid-3TMS	13.043	2.6
42	Methionine-2TMS	13.047	2.8
43	5-Oxoproline-2TMS	13.151	3.05
44	2-Hydroxyglutaric acid-3TMS	13.444	2.65
45	Creatinine enol-3TMS	13.446	2.75

46	2-ketoglutaric acid-methyloxime-2TMS	13.549	2.95
47	Phenylalanine-TMS	13.554	3.25
48	2-Phosphoglycolic acid-3TMS	13.748	2.9
49	Tiglyglycine-TMS	13.855	3.3
50	Glutamic acid-3TMS	14.044	2.65
51	2-Pyrrolidone-5-carboxylic acid-TMS	14.053	3.15
52	Tartaric acid-4TMS	14.243	2.55
53	D-Lyxose-methyloxime-4TMS	14.342	2.5
54	Phenylalanine-2TMS	14.348	2.85
55	Lauric acid-TMS	14.545	2.7
56	Pyrophosphoric acid-4TMS	14.55	3.0
57	*N*-Acetylaspartic acid-2TMS	14.652	3.1
58	Asparagine-3TMS	14.747	2.8
59	Taurine-3TMS	14.848	2.85
60	Putrescine-4TMS	15.442	2.5
61	3-Phosphoglycerol-4TMS	15.544	2.65
62	Quinolinic acid-2TMS	15.655	3.3
63	D-Xylopyranose-5TMS	15.841	2.45
64	2-Aminoethyl phosphate-4TMS	15.846	2.75
65	Glutamine-3TMS	15.95	3.2
66	N-Acetylglutamic acid-2TMS	16.053	3.15
67	Citric acid-4TMS	16.144	2.65
68	Citrulline-4TMS/Arginine-4TMS	16.346	2.8
69	D-Galactofuranose-6TMS	16.447	2.8
70	Cys-gly-TMS	16.565	3.9
71	Myristic acid-d3-TMS	16.746	2.75
72	D-Glucose-methyloxime-5TMS (1Z)	16.842	2.5
73	α-Methyl glycoside-4TMS	16.943	2.45
74	D-Talose-methyloxime-5TMS (anti)	17.042	2.5
75	Hippuric acid-TMS	17.06	3.6
76	D-Sorbitol-6TMS	17.24	2.4
77	Lysine-4TMS	17.243	2.6
78	Tyrosine-2TMS	17.252	3.1
79	Gluconic acid-6TMS	17.441	2.45
80	L-Ascorbic acid-4TMS	17.446	2.75
81	Histidine-3TMS	17.451	3.05
82	D-Talopyranose-5TMS	17.541	2.45
83	Tyrosine-3TMS	17.547	2.8
84	Methyl Palmitate	17.648	2.95
85	Hexadecanol-TMS	17.744	2.65
86	n-Pentadecanoic acid-TMS	17.746	2.75
87	Gulonic acid-1,4-lactone-4TMS	17.843	2.55
88	Pantothenic acid-3TMS	18.047	2.8
89	*Scyllo*-Inositol-6TMS	18.142	2.5
90	Palmitelaidic acid-TMS	18.448	2.85
91	7H-Purine-3TMS	18.56	3.6
92	Palmitic acid-TMS	18.647	2.8
93	*Myo*-Inositol-6TMS	18.742	2.5
94	Galactose-5TMS-trimethyloxime	19.042	2.5
95	2-Propenoic acid, pentadecyl ester	19.249	2.95
96	Methyl Oleate	19.351	3.05

97	Octadec-9Z-enol-TMS	19.446	2.75
98	*cis*-10-Heptadecenoic acid-TMS	19.448	2.85
99	Methyl stearate	19.549	2.95
100	Margaric acid-TMS	19.65	3.0
101	Palmidrol	19.753	3.2
102	Oleic acid-TMS	20.248	2.85
103	Hexadecanamide	20.257	3.4
104	Tryptophan-2TMS	20.358	3.5
105	Spermidine-5TMS	20.443	2.55
106	Stearic acid-TMS	20.447	2.8
107	m-Hydroxyhippuric acid-2TMS	20.555	3.3
108	D-Ribose-methyloxime-5-phosphate-5TMS	20.644	2.65
109	D-Glucose-methyloxime-6-phosphate-6TMS	20.744	2.70
110	Methyl arachidonate	20.853	3.2
111	D-Mannopyranose-6-phosphate-6TMS	21.243	2.75
112	Monomyristoylglycerol-2TMS	21.645	2.7
113	Arachidonic acid-TMS	21.649	2.95
114	*cis*-5,8,11-Eicosatrienoic acid-TMS	21.749	2.95
115	5-MethylUridine-TMS	21.752	3.1
116	11,14-Eicosadienoic acid-TMS	21.848	2.95
117	Oleamide	21.858	3.45
118	11-Eicosenoic acid, trimethylsilyl ester	21.948	2.85
119	1-O-hexadecylglycerol-2TMS	22.245	2.7
120	Uridine-3TMS	22.252	3.1
121	Nonyl 2-*N,N*-dimethylaminoethyl fumarate	22.351	3.05
122	Methyl 4,7,10,13,16,19-docosahexaenoate	22.456	3.35
123	2-Monopalmitoylglycerol-2TMS	22.846	2.75
124	Inosine-4TMS	23.051	3.05
125	Dioctyl phthalate	23.058	3.45
126	Monopalmtin-2TMS	23.146	2.75
127	*cis*-4,7,10,13,16,19-Docosahexaenoic acid-TMS	23.152	3.1
128	Methyl 7,10,13,16-docosatetraenoate	23.163	3.75
129	Maltose-8TMS (isomer 1)	23.343	2.55
130	Adenosine-4TMS	23.55	3
131	Maltose-8TMS (isomer 2)	23.642	2.50
132	1-Octadecylglycerol-2TMS	23.646	2.75
133	Maltose-8TMS-Methyloxime (isomer 1)	23.842	2.50
134	Maltose-8TMS-Methyloxime (isomer 2)	24.043	2.55
135	2-Monostearin-2TMS	24.247	2.8
136	1-Monooleoylglycerol-2TMS	24.348	2.85
137	Guanosine-5TMS	24.451	3.05
138	Monostearin-2TMS	24.547	2.8
139	Dioctyl isophthalate	24.653	3.2
140	5'-S-Methylthioadenosine-3TMS	24.756	3.4
141	Uridine-5'-monophosphate-5TMS	24.854	3.25
142	*cis*-15-Tetracosenoic acid-TMS	24.948	2.9
143	Tetracosanoic acid-TMS	25.048	2.85
144	Squalene	25.058	3.15
145	Monoarachidoylglycerol-2TMS	25.847	2.75
146	Cholesta-3,5-diene	26.057	3.35
147	Adenosine-5'-monophosphate-5TMS	26.148	3.2

148	Guanosine-5'-monophosphate-6TMS	26.563	3.7
149	Tocopherol-TMS	27.256	3.30
150	Cholesterol-TMS	27.66	3.55
151	Desmosterol-TMS	27.962	3.65
152	Lactose-8TMS	28.048	2.80
153	5α-Cholestan-3β-ol-TMS	28.27	4.05
154	Cholan-24-oic acid-4TMS	28.357	3.35
155	Campesterol-TMS	28.463	3.7
156	Palmityl palmitate	29.061	3.60
157	Cellobiose-8TMS (isomer 2)	29.148	2.85
158	Sitosterol-TMS	29.265	3.85
159	Lanosterol-TMS	29.368	4
160	Cellobiose-methyloxime-8TMS (isomer 1)	29.749	2.90
161	Cellobiose-methyloxime-8TMS (isomer 2)	30.150	2.95
162	1,3-Dimyristoylglycerol-1TMS	30.768	4.05
163	Oleyl palmitate	31.173	4.35
164	Stearyl palmitate	31.373	4.30
165	1,3-Dipalmitin-TMS	33.583	4.85

155 metabolites from 165 features
